# Role of Forkhead Transcription Factors in Diabetes-Induced Oxidative Stress

**DOI:** 10.1155/2012/939751

**Published:** 2012-01-31

**Authors:** Bhaskar Ponugoti, Guangyu Dong, Dana T. Graves

**Affiliations:** School of Dental Medicine, University of Pennsylvania, Philadelphia, PA 19104-6030, USA

## Abstract

Diabetes is a chronic metabolic disorder, characterized by hyperglycemia resulting from insulin deficiency and/or insulin resistance. Recent evidence suggests that high levels of reactive oxygen species (ROS) and subsequent oxidative stress are key contributors in the development of diabetic complications. The FOXO family of forkhead transcription factors including FOXO1, FOXO3, FOXO4, and FOXO6 play important roles in the regulation of many cellular and biological processes and are critical regulators of cellular oxidative stress response pathways. FOXO1 transcription factors can affect a number of different tissues including liver, retina, bone, and cell types ranging from hepatocytes to microvascular endothelial cells and pericytes to osteoblasts. They are induced by oxidative stress and contribute to ROS-induced cell damage and apoptosis. In this paper, we discuss the role of FOXO transcription factors in mediating oxidative stress-induced cellular response.

## 1. Introduction

Diabetes mellitus is a chronic disease characterized by elevated blood sugar levels resulting from either lack of insulin production or resistance to insulin. In 2010, there were nearly 230 million individuals with diabetes worldwide which is estimated to reach 430 million by 2030 [1]. Recently, a study conducted by the U.S. Centers for Disease Control and Prevention (CDC) indicated that 25.8 million Americans or 8.3% of its population were affected by diabetes in 2010 [2]. Diabetes has severe health consequences associated with numerous diabetic complications including retinopathy, neuropathy, and nephropathy [3–5]. Accumulating evidence suggests that hyperglycemia-induced production of free radicals and the subsequent oxidative stress contributes to the development and progression of diabetes and related complications [6–8].

Reactive oxygen species (ROS) are oxygen free radicals that are generated as by-products of mitochondrial metabolism and function as signaling molecules in various intracellular processes including cell proliferation, migration, and apoptosis [9]. ROS produced during normal metabolic processes are removed rapidly with the help of various endogenous detoxifying enzymes. While normal cellular ROS concentrations are necessary for proper functioning of cells, excessive, non-physiological concentrations of ROS result in oxidative stress. ROS such as superoxide (O_2_^−^) and hydroxyl radicals (HO^•^), and hydrogen peroxide (H_2_O_2_), are highly reactive and can cause damage to biological macromolecules such as DNA, proteins, and lipids [9]. Major sources of oxidative stress during diabetes include glucose autooxidation, overproduction of ROS by mitochondria, non-enzymatic glycation, and the polyol pathway [6, 10]. In the polyol pathway, aldose reductase converts glucose into sorbitol with NADPH as a coenzyme. In diabetes, increased flux through the polyol pathway enhances oxidative stress because of increased consumption of NADPH by aldose reductase. Since NADPH is required for generation of endogenous antioxidant glutathione (GSH), reduced NADPH availability depletes GSH leading to greater oxidative stress [6]. Other mechanisms through which high glucose levels can lead to advanced glycation endproducts are discussed below.

ROS leads to the generation of intracellular signals that stimulate inflammation and cell death. They include protein kinase C (PKC), c-Jun-N-terminal kinase (JNK), and p38 mitogen-activated protein kinase (MAPK) [11–15]. In many cell types, ROS lead to the activation of the forkhead box O (FOXO) transcription factors that include FOXO1, FOXO3, and FOXO4, which can mediate the effects of ROS through regulation of gene transcription. These transcription factors have been implicated in diverse cellular processes ranging from glucose metabolism to cell behavior including cell cycle and apoptosis [16, 17]. In addition to being activated by ROS, FOXO proteins play a critical role in oxidative stress by upregulating expression of antioxidant genes [9]. However, FOXO proteins are involved in many other processes and can have apparently contradictory effects in different cell types [18]. FOXO proteins are transcription factors but also have important function as corepressors or coactivators so that direct DNA binding is not a prerequisite for modulating the transcription of gene targets [19]. For simplicity, we will use the term FOXO for all or any of the FOXO transcription factors throughout this paper, unless otherwise specified.

## 2. Regulation of FOXO by Oxidative Stress

FOXO transcription factors are critical mediators of oxidative stress and are activated by various kinds of cellular stress stimulus. Oxidative stress regulates FOXO activity through various posttranslational modifications including phosphorylation, acetylation, and ubiquitination, which in turn regulate the subcellular localization of FOXOs, protein-protein interactions, and transcriptional activity of FOXO proteins. While some of these modifications promote FOXO transcriptional activity, others are inhibitory. For example, stress-activated kinase JNK directly phosphorylates FOXO4 at residues Thr447 and Thr451, which leads to its nuclear translocation and induces FOXO4 transcriptional activity [20]. Another kinase implicated in oxidative stress-induced phosphorylation of FOXO is mammalian Ste20-like protein kinase 1 (MST1). During oxidative stress, MST1 phosphorylates FOXO3 at residue Ser207, which results in FOXO3 release from binding protein, 14-3-3. This release allows FOXO3 to translocate to the nucleus thereby modulating target gene expression [21].

FOXO transcriptional activity is also regulated by acetylation. The effects of oxidative stress-induced acetylation on FOXO function vary based upon the experimental conditions. Sirtuins (SIRTs), mammalian homologs of the yeast silent information regulator 2 (sir2) deacetylase, are critical regulators of FOXO transcriptional activity and are induced by oxidative stress [22, 23]. It has been reported that acetylation by cAMP-response-element-binding-protein (CREB-) binding protein (CBP)/P300 positively regulates FOXO transcriptional activity during oxidative stress, while SIRT1-mediated deacetylation represses the activity of FOXO transcription factors (FOXO1, FOXO3, and FOXO4) [24]. Other reports suggest that oxidative stress-induced FOXO4 acetylation negatively regulates its transcriptional activity, and deacetylation by SIRT1 counteracts the acetylation-mediated FOXO4 inhibition [25]. Furthermore, studies from Brunet et al. suggest that SIRT1 differentially affects FOXO3 function in response to oxidative stress [23]. SIRT1 associates with and deacetylates FOXO3 both *in vitro* and *in vivo*. SIRT1 deacetylation of FOXO3 increases expression of its target genes involved in cell cycle arrest and DNA repair such as p27 and GADD45. In contrast, SIRT1 deacetylation reduces expression of FOXO3 proapoptotic target genes such Bim and Fas ligand. These results indicate that deacetylation can both enhance and reduce FOXO3-induced activity depending upon the target gene.

**Figure 1:**
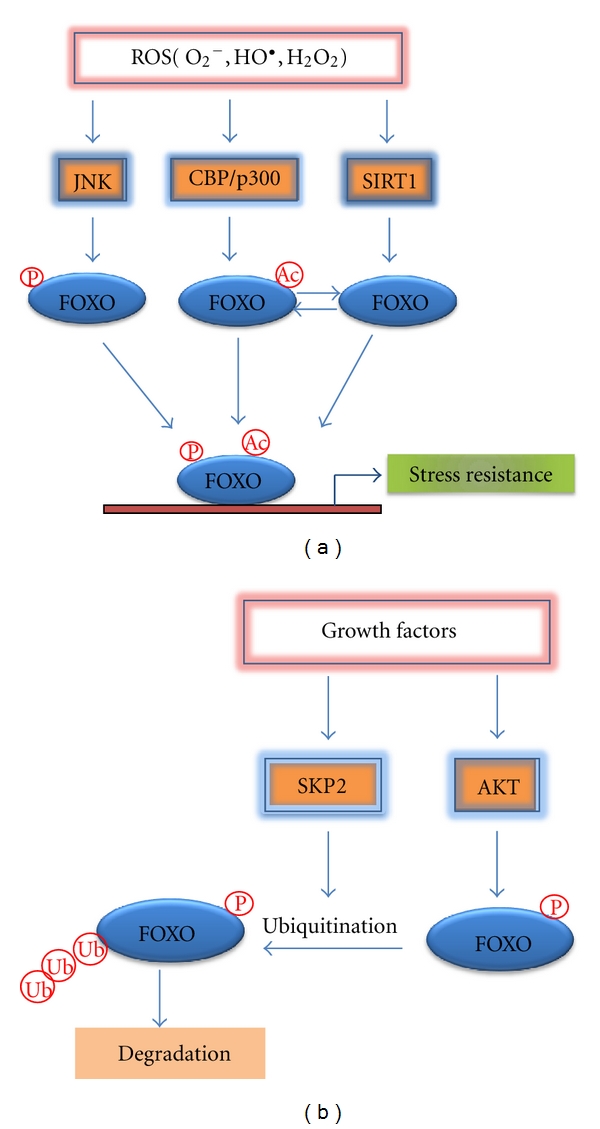
(a) Model of FOXO regulation during ROS-induced oxidative stress. In response to ROS-induced oxidative stress, the activity of FOXO proteins is modulated by various posttranslational modifications including phosphorylation and acetylation. The stress-activated kinase JNK phosphorylates FOXO leading to its nuclear translocation and activation. FOXO is acetylated by acetyltransferase CBP/p300 upon oxidative stress stimuli and deacetylated by SIRT1 deacetylase. Change in the acetylation status may activate or inhibit FOXO activity depending on the target genes and experimental conditions. Activation of FOXO by various posttranslational modifications leads to the induction of stress response genes such as MnSOD, catalase, and GADD45*α*. (b) Negative regulation of FOXO by growth factor signaling. Upon growth factor stimulation, AKT phosphorylates FOXO proteins on conserved residues, leading to their nuclear exclusion. SKP2-dependent ubiquitination, which may be induced by Akt, leads to its subsequent degradation. P: phosphorylation; Ac: acetylation; Ub: ubiquitination.

Besides phosphorylation and acetylation, FOXO proteins are further regulated by ubiquitination during oxidative stress. In response to insulin or growth factor signaling, FOXO transcription factors are phosphorylated, polyubiquitinated, and degraded [26]. It has been reported that AKT-dependent phosphorylation is required as a prerequisite for ubiquitin-mediated degradation of FOXO1 and FOXO3. FOXO ubiquitination is mediated by F-box protein Skp2, a subunit of the SCF (Skp1/Cul1/F-box) E3 ubiquitin ligase protein complex [27, 28]. In contrast to insulin/growth factor signaling, upon oxidative stress, FOXO4 becomes monoubiquitinated and translocated into the nucleus, resulting in its increased transcriptional activity. Monoubiquitination of FOXO4 is mediated by E3 ubiquitin ligase murine double minute 2 (MDM2) [28].  Figure 1(a) shows the effect of ROS-induced oxidative stress that regulates FOXO by altering its phosphorylation or acetylation status. In contrast, growth factor-mediated induction of AKT which phosphorylates FOXO at specific amino acids leads to its export from the nucleus and Skp2 which leads to its ubiquitination and degradation (Figure 1(b)).

## 3. Role of FOXO in Oxidative Stress

FOXO proteins play an important role in protection of cells against oxidative stress. Oxidative stress is caused by overproduction of ROS or inefficient breakdown of ROS. Efficient detoxification of ROS by cellular detoxification systems protects cells against oxidative damage. The levels and enzymatic activities of various antioxidant enzymes such as manganese superoxide dismutase (MnSOD), catalase, and glutathione peroxidase are decreased during hyperglycemia-induced oxidative stress [11]. It is now well established that cells activate FOXO transcription factors to reduce the level of oxidative stress by the induction of enzymes that breakdown ROS such as MnSOD and catalase [29, 30]. For example, FOXO3 directly binds to MnSOD promoter at FOXO binding elements to increase its expression. Activation of MnSOD in mitochondria protects cells from ROS-mediated injury by converting superoxide radicals to oxygen and hydrogen peroxide (H_2_O_2_). Enzymes catalase and glutathione peroxidase further breakdown H_2_O_2_ into water and oxygen [30, 31]. The functional significance of FOXO in regulating oxidative stress is further revealed by gene deletion studies. Mice lacking FOXO factors (FOXO 1/3/4) in hematopoietic stem cells (HSCs) exhibit decreased self-renewal, leading to defective repopulating activity [32]. Consistent with this, FOXO-deficient HSCs showed increased ROS levels, decreased expression of antioxidant proteins, and increased apoptosis, suggesting critical role of FOXOs in stress resistance. Recent evidence suggests that FOXO factors play a fundamental role in skeletal homeostasis by upregulating antioxidant enzymes [33, 34]. Deletion of FOXO1 in osteoblasts results in decreased expression of antioxidants such as glutathione. The resulting increased oxidative stress reduces osteoblast numbers and bone formation. Consistent with this, conditional deletion of FOXO factors (FOXO 1/3/4) in bone results in increased oxidative stress, loss of osteoblasts, and decreased bone mass, suggesting, FOXO factors are indispensable for skeletal homeostasis because of their antioxidant defense properties [33, 34]. It was also found that FOXO1 deletion in osteoblasts is associated with decreased protein synthesis. FOXO1 promotes protein synthesis in osteoblasts through direct regulation of ATF4, a transcription factor required of amino acid import and protein synthesis [33]. In the previous example, FOXO1 is protective through the induction of antioxidants. However, under conditions where inflammation is high, FOXO1 may also have a direct effect on osteoblasts by mediating inflammation-induced apoptosis [35]. In this case, FOXO1 is thought to induce expression of proapoptotic factors and exert an apoptotic rather than a protective effect. Under conditions of bone formation, FOXO1 may exert another set of effects. It has been reported that FOXO1 is needed for differentiation of osteoblast precursors to osteoblasts and that overexpression of FOXO1 interferes with progression of osteoblast precursors through the cell cycle [36]. Thus, the impact of FOXO1 on osteoblasts or their precursors may be highly dependent upon the context and microenvironment.

## 4. Role of FOXO in Cell Proliferation and Survival

FOXO transcription factors play a role in cell proliferation and survival by regulating the expression of genes involved in a number of cellular processes including cell cycle arrest, DNA repair, and apoptosis. In response to certain levels of oxidative stress, FOXO factors induce expression of target genes that control cell cycle progression and DNA repair, including p27Kip1, retinoblastoma-like protein p130, and cyclin D1/2, growth arrest, and DNA damage-inducible gene 45*α* (GADD45*α*) [37–41]. For example, FOXO causes cell-cycle arrest in G1 phase by inducing negative cell-cycle regulators such as cdk inhibitor p27^kip1^ [37] and by repressing the expression of G1 cyclins D1 and D2 [40]. Besides promoting cell cycle arrest, FOXO also plays a critical role in stress resistance by facilitating repair of damaged DNA. FOXO3 induces cell cycle arrest at G2-M checkpoint and triggers DNA repair by inducing expression of the DNA damage response gene GADD45*α* [41]. Although in most cases FOXO proteins are associated with cell cycle arrest, in some cases FOXO proteins appear to promote cell cycle progression.

FOXO transcription factors typically induce either cell death by regulating proapoptotic genes but depending upon the context can enhance survival. In response to certain ROS levels, FOXO transcription factors switch from prosurvival to proapoptotic signaling leading to cell death. However, the exact molecular mechanisms by which FOXO switches from prosurvival to prodeath signaling remain unknown. In diabetes, chronic hyperglycemia-induced mitochondrial ROS stimulate various signaling pathways leading to activation of FOXO, which in turn activates several proapoptotic factors. FOXO1 activation is elevated in diabetic connective tissue and mediates advanced glycation endproduct and TNF-alpha-induced apoptosis both of which are elevated in diabetic connective tissue [42–44]. It has been proposed that diabetes-enhanced activation of FOXO1 limits wound healing by enhancing fibroblast apoptosis and proliferation [43]. FOXO1 regulates genes of both the extrinsic and intrinsic apoptotic pathways [42]. FOXO3 and FOXO4 induce apoptosis by directly binding Bcl-6 promoter and enhancing its expression and negatively regulate expression of an antiapoptotic protein BCL-X_L_ [45]. It was further shown that silencing endogenous FOXO3 or overexpression of a dominant negative mutant of FOXO3 resulted in decreased expression of a variety of proapoptotic genes, including Bcl-6 and Bim, in response to hydrogen peroxide-induced oxidative stress. We have recently shown that hyperglycemia during diabetes stimulates microvascular endothelial cell and pericyte apoptosis leading to early stages of diabetic retinopathy [46]. High glucose leads to ROS generation that enhances FOXO1 activation and induction of several classes of genes that regulate endothelial cell behavior including proapoptotic and proinflammatory factors. These results suggest that FOXO1 plays an important role in the development of diabetic retinopathy due to its effect on inflammatory and apoptotic gene expression in microvascular cells [46]. Moreover, high glucose and advanced glycation endproducts that are elevated in diabetes stimulate loss of microvascular retinal pericytes through a process that involves activation of FOXO1 [46, 47]. In the latter, advanced glycation endproducts activate FOXO1 in pericytes through the MAP kinase pathway, and the loss of pericytes is countered by activation of Akt and NF-kappaB [47].

## 5. FOXO in Diabetes-Induced Inflammation

Inflammation has long been considered as a major risk factor in diabetes and associated with development and progression of diabetic complications. Hyperglycemia-induced oxidative stress promotes inflammation through increased endothelial cell damage, microvascular permeability, and increased release of proinflammatory cytokines, including TNF-*α*, interlukin-1*β* (IL-1*β*), and interlukin-6 (IL-6), ultimately leading to decreased insulin sensitivity and diabetic complications. Hyperglycemia-induced FOXO plays an important role in the induction of proinflammatory cytokines. It was shown that FOXO1 directly binds to IL-1*β* promoter and increases its expression in macrophages [48]. FOXO1 is induced by inflammatory cytokines and may be involved in a forward amplification loop. For example, in microvascular endothelial cells, FOXO1 is induced *in vivo* by diabetes-enhanced TNF-*α* and also induces expression of TNF-*α* levels in these cells [46]. Increased IL-1*β* and TNF-*α* production has been implicated in pathogenesis of obesity and diabetes. Hyperglycemia in diabetes also stimulates toll-like receptor (TLR) signaling, which results in prolonged inflammation and tissue damage. Recent studies show that FOXO1 promotes inflammation during diabetes by enhancing TLR4-mediated signaling, suggesting FOXO1 as a key mediator of inflammatory responses during obesity and diabetes [49]. In diabetic fracture healing, there is enhanced upregulation of proinflammatory and proapoptotic factors [50, 51]. It has been shown that FOXO1 induces expression of both proinflammatory and proapoptotic factors in chondrocytes and that FOXO1 directly binds to the TNF-*α* promoter. Moreover, diabetes-enhanced TNF-*α* activates FOXO1 in chondrocytes *in vivo* by enhancing its nuclear localization [50].

Another transcription factor that plays an important role in stimulating inflammation during hyperglycemia and oxidative stress is NF-*κ*B [52]. Activation of NF-*κ*B pathway has been implicated in the development of diabetic complications, including retinopathy, and has been shown to regulate expression of various proinflammatory cytokines, including TNF-*α* and IL-1*β* [53]. Chronically elevated ROS levels associated with diabetes may induce both NF-*κ*B and FOXO leading to increased inflammation and cellular damage. In most cell types, NF-*κ*B is directly antiapoptotic, while FOXO1 is directly proapoptotic. Thus, in inflammatory conditions when both NF-*κ*B and FOXO1 are activated, their relative balance may determine whether a cell ultimately survives or undergoes apoptosis [42, 47].

### 5.1. Mitochondria, ROS, and Diabetes

A mechanism through which diabetes can increase oxidative stress involves electron transport in mitochondria. It has been proposed that high intracellular glucose levels increase the follow of electrons through the electron transport chain in mitochondria during oxidative respiration [6]. This can result in the transfer of electrons to O_2_ leading to formation of O_2_^−^ and the generation of various reactive oxygen species in the mitochondria. Furthermore, changes caused by diabetes alter the redox balance and affect redox-sensitive proteins such as protein kinase C-epsilon, which can result in enhanced mitochondrial ROS production. Advanced glycation end products (AGEs) generated under conditions of hyperglycemia stimulate NADPH oxidase that in turn can induce production of ROS. In a surprising development, increased Wnt signaling stimulates mitochondrial biogenesis that can lead to enhanced ROS levels in mitochondria and greater oxidative damage [54]. The increased ROS in mitochondria is thought to be problematic due to a number of different mechanisms. One is that ROS damages mitochondrial components such as DNA, membrane proteins, and lipids. ROS can also induce the opening of the mitochondrial permeability transition pore (MPTP) [55]. When this pore is opened, proapoptotic proteins are released from the mitochondria such as cytochrome c that stimulate cell death. ROS generated in the mitochondrial respiratory chain have been proposed as secondary messengers for activation of NF-*κ*B by TNF-*α* and IL-1 [6].

ROS may affect insulin signalling. Insulin signalling is reduced under conditions of oxidative stress, which may contribute to insulin resistance. This may occur through several mechanisms. In one scenario, ROS induces serine phosphorylation of insulin receptor substrate, decreasing tyrosine phosphorylation thereby interfering with insulin signaling [56]. Similarly, ROS have been shown to partially mediate the effect of Angiotensin II inhibition of insulin signalling [57]. Methylglyoxal, a biologically active AGE precursor formed under conditions of hyperglycemia, inhibits phosphorylation of insulin receptor substrate and activation of the phosphatidylinositol 3-kinase (PI3K)/protein kinase B (PKB) pathway [58].

**Figure 2:**
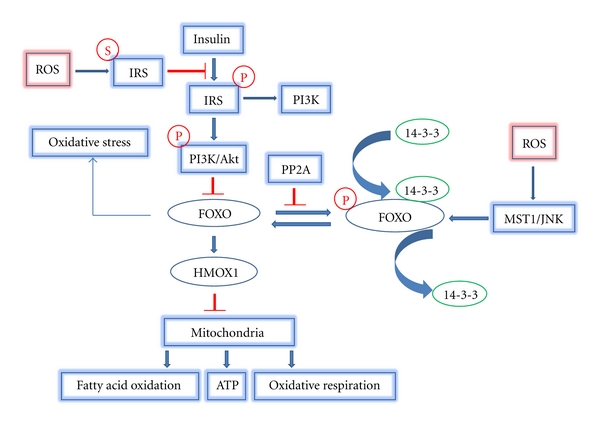
Oxidative stress and insulin signaling affect mitochondrial function via FOXO. ROS induce IRS serine phosphorylation which inhibits IRS activation by insulin signalling. As a result of reduced IRS activity, Akt activity is reduced. Reduced Akt reduces negative signalling of FOXO so that FOXO1 is left in an activated state since it is not exported out of the nucleus by 14-3-3. Meanwhile, ROS activate MST1 and JNK which induce FOXO nuclear translocation by disrupting the complex of FOXO and 14-3-3. PP2A activates FOXO by dephosphorylation of FOXO and by Akt dephosphorylation. FOXO nuclear translocation will induce HMOX1 gene expression which inhibits mitochondrial function by affecting like fatty acid oxidation, ATP, and oxidative respiration (arrow indicates stimulatory event; bar indicates inhibitory event).

 Insulin signalling inactivates FOXO1, which is mediated by insulin receptor substrates-1 and -2 through AKT. A characteristic feature of insulin resistance is the elevated production of glucose that contributes to hyperglycemia. FOXO1 regulates glucose production in the liver through the expression of genes that promote gluconeogenesis [59]. Thus, a pathway exists whereby insulin resistance leads to elevated FOXO1 activation, upregulation of genes that promote glucose production, and greater serum glucose levels. Disruption of the insulin-Akt-FOXO1 balance also affects the mitochondria. Activated FOXO1 induces heme oxygenase-1 (HMOX1), which cleaves heme and disrupts the mitochondrial electron transport chain [60]. Thus, when FOXO1 activity is elevated by insulin resistance, greater expression of heme oxygenase-1 ensues. Greater heme oxygenase-1 levels interfere with mitochondria leading to impaired oxidative respiration, negatively affecting fatty acid oxidation and the production of ATP. Furthermore, enhanced activation of FOXO1 affects the expression mitochondrial fusion and fission thereby affecting mitochondrial biogenesis. Under conditions of insulin resistance, there are insufficient mitochondria and abnormal mitochondrial morphology, which is reversed when FOXO1 is deleted [60].  Figure 2 demonstrates the complex signalling pathways through which oxidative stress and insulin can modulate FOXO activity to affect mitochondria.

## 6. Conclusion and Perspective

Hyperglycemia-induced ROS and subsequent oxidative stress are major contributors to the development and progression of diabetes and related complications. However, effective therapeutic strategies to prevent the generation of these free radicals remain limited. It is now well established that FOXO transcription factors are the critical regulators of cell fate and play a major role in diabetes-induced oxidative stress resistance and in diabetes-enhanced apoptosis. It seems that FOXO transcription factors might function as molecular switches that determine cell fate in response to various levels of oxidative stress by either promoting antioxidants (prosurvival) responses or alternatively enhancing proapoptotic gene expression and cell death. However, the precise mechanism by which FOXO mediates prosurvival/proapoptotic response remains unclear and elucidation of molecular mechanisms involved may provide new targets for therapy. Furthermore, multiple signaling pathways, including JNK and MAPK, regulate the activity of FOXO transcription factors in response to hyperglycemia in diabetes. Because our understanding of how these diverse signaling pathways coordinate their effects to regulate FOXO activity during diabetes remains limited, detailed understanding of these pathways may provide insights into development of new therapeutic strategies for treatment of diabetes.

## References

[1] IDF (2009). Diabetes and impaired glucose tolerance. Global burdan: prevalance and projections 2010 and 2030. http://www.idf.org/diabetesatlas/diabetes-and-impaired-glucose-tolerance.

[2] CDC National diabetes fact sheet: national estimates and general information on diabetes and prediabetes in the United States. http://www.cdc.gov/diabetes/pubs/factsheet11.htm.

[3] Calcutt NA, Cooper ME, Kern TS, Schmidt AM (2009). Therapies for hyperglycaemia-induced diabetic complications: from animal models to clinical trials. *Nature Reviews Drug Discovery*.

[4] Shamoon H, Duffy H, Fleischer N (1993). The effect of intensive treatment of diabetes on the development and progression of long-term complications in insulin-dependent diabetes mellitus. *The New England Journal of Medicine*.

[5] Singh DK, Winocour P, Farrington K (2011). Oxidative stress in early diabetic nephropathy: fueling the fire. *Nature Reviews Endocrinology*.

[6] Giacco F, Brownlee M (2010). Oxidative stress and diabetic complications. *Circulation Research*.

[7] Rains JL, Jain SK (2011). Oxidative stress, insulin signaling, and diabetes. *Free Radical Biology and Medicine*.

[8] Pitocco D, Zaccardi F, di Stasio E (2010). Oxidative stress, nitric oxide, and diabetes. *Review of Diabetic Studies*.

[9] Storz P (2011). Forkhead homeobox type O transcription factors in the responses to oxidative stress. *Antioxidants and Redox Signaling*.

[10] Wohaieb SA, Godin DV (1987). Alterations in free radical tissue-defense mechanisms in streptozocin-induced diabetes in rat. Effects of insulin treatment. *Diabetes*.

[11] Nishikawa T, Kukidome D, Sonoda K (2007). Impact of mitochondrial ROS production on diabetic vascular complications. *Diabetes Research and Clinical Practice*.

[12] Sivitz WI, Yorek MA (2010). Mitochondrial dysfunction in diabetes: from molecular mechanisms to functional significance and therapeutic opportunities. *Antioxidants and Redox Signaling*.

[13] Basta G, Schmidt AM, de Caterina R (2004). Advanced glycation end products and vascular inflammation: implications for accelerated atherosclerosis in diabetes. *Cardiovascular Research*.

[14] Thomas SR, Witting PK, Drummond GR (2008). Redox control of endothelial function and dysfunction: molecular mechanisms and therapeutic opportunities. *Antioxidants and Redox Signaling*.

[15] Marshall S, Garvey WT, Traxinger RR (1991). New insights into the metabolic regulation of insulin action and insulin resistance: role of glucose and amino acids. *FASEB Journal*.

[16] Myatt SS, Lam EWF (2007). The emerging roles of forkhead box (Fox) proteins in cancer. *Nature Reviews Cancer*.

[17] Ho KK, Myatt SS, Lam EWF (2008). Many forks in the path: cycling with FoxO. *Oncogene*.

[18] Paik JH, Kollipara R, Chu G (2007). FoxOs are lineage-restricted redundant tumor suppressors and regulate endothelial cell homeostasis. *Cell*.

[19] van der Vos KE, Coffer PJ (2008). FOXO-binding partners: it takes two to tango. *Oncogene*.

[20] Essers MAG, Weijzen S, de Vries-Smits AMM (2004). FOXO transcription factor activation by oxidative stress mediated by the small GTPase Ral and JNK. *EMBO Journal*.

[21] Lehtinen MK, Yuan Z, Boag PR (2006). A conserved MST-FOXO signaling pathway mediates oxidative-stress responses and extends life span. *Cell*.

[22] Guarente L (2011). Sirtuins, aging, and medicine. *The New England Journal of Medicine*.

[23] Brunet A, Sweeney LB, Sturgill JF (2004). Stress-dependent regulation of FOXO transcription factors by the SIRT1 deacetylase. *Science*.

[24] Motta MC, Divecha N, Lemieux M (2004). Mammalian SIRT1 represses forkhead transcription factors. *Cell*.

[25] van der Horst A, Tertoolen LGJ, de Vries-Smits LMM, Frye RA, Medema RH, Burgering BMT (2004). FOXO4 is acetylated upon peroxide stress and deacetylated by the longevity protein hSir2(SIRT1). *Journal of Biological Chemistry*.

[26] de Keizer PLJ, Burgering BMT, Dansen TB (2011). Forkhead box O as a sensor, mediator, and regulator of redox signaling. *Antioxidants and Redox Signaling*.

[27] Huang H, Regan KM, Wang F (2005). Skp2 inhibits FOXO1 in tumor suppression through ubiquitin-mediated degradation. *Proceedings of the National Academy of Sciences of the United States of America*.

[28] Huang H, Tindall DJ (2011). Regulation of FOXO protein stability via ubiquitination and proteasome degradation. *Biochimica et Biophysica Acta*.

[29] Kops GJPL, Dansen TB, Polderman PE (2002). Forkhead transcription factor FOXO3a protects quiescent cells from oxidative stress. *Nature*.

[30] Nemoto S, Finkel T (2002). Redox regulation of forkhead proteins through a p66shc-dependent signaling pathway. *Science*.

[31] van der Horst A, Burgering BMT (2007). Stressing the role of FoxO proteins in lifespan and disease. *Nature Reviews Molecular Cell Biology*.

[32] Tothova Z, Kollipara R, Huntly BJ (2007). FoxOs are critical mediators of hematopoietic stem cell resistance tophysiologic oxidative stress. *Cell*.

[33] Rached MT, Kode A, Xu L (2010). FoxO1 is a positive regulator of bone formation by favoring protein synthesis and resistance to oxidative stress in osteoblasts. *Cell Metabolism*.

[34] Ambrogini E, Almeida M, Martin-Millan M (2010). FoxO-mediated defense against oxidative stress in osteoblasts is indispensable for skeletal homeostasis in mice. *Cell Metabolism*.

[35] Behl Y, Siqueira M, Ortiz J (2008). Activation of the acquired immune response reduces coupled bone formation in response to a periodontal pathogen. *Journal of Immunology*.

[36] Siqueira MF, Flowers S, Bhattacharya R (2011). FOXO1 modulates osteoblast differentiation. *Bone*.

[37] Medema RH, Kops GJPL, Bos JL, Burgering BMT (2000). AFX-like forkhead transcription factors mediate cell-cycle regulation by Ras and PKB through p27(kip1). *Nature*.

[38] Furukawa-Hibi Y, Yoshida-Araki K, Ohta T, Ikeda K, Motoyama N (2002). FOXO forkhead transcription factors induce G2-M checkpoint in response to oxidative stress. *Journal of Biological Chemistry*.

[39] Nakamura N, Ramaswamy S, Vazquez F, Signoretti S, Loda M, Sellers WR (2000). Forkhead transcription factors are critical effectors of cell death and cell cycle arrest downstream of PTEN. *Molecular and Cellular Biology*.

[40] Schmidt M, de Mattos SF, van der Horst A (2002). Cell cycle inhibition by FoxO forkhead transcription factors involves downregulation of cyclin D. *Molecular and Cellular Biology*.

[41] Tran H, Brunet A, Grenier JM (2002). DNA repair pathway stimulated by the forkhead transcription factor FOXO3a through the Gadd45 protein. *Science*.

[42] Alikhani M, Alikhani Z, Graves DT (2005). FOXO1 functions as a master switch that regulates gene expression necessary for tumor necrosis factor-induced fibroblast apoptosis. *Journal of Biological Chemistry*.

[43] Siqueira MF, Li J, Chehab L (2010). Impaired wound healing in mouse models of diabetes is mediated by TNF-*α* dysregulation and associated with enhanced activation of forkhead box O1 (FOXO1). *Diabetologia*.

[44] Alikhani M, MacLellan CM, Raptis M, Vora S, Trackman PC, Graves DT (2007). Advanced glycation end products induce apoptosis in fibroblasts through activation of ROS, MAP kinases, and the FOXO1 transcription factor. *American Journal of Physiology—Cell Physiology*.

[45] Tang TTL, Dowbenko D, Jackson A (2002). The forkhead transcription factor AFX activates apoptosis by induction of the BCL-6 transcriptional repressor. *Journal of Biological Chemistry*.

[46] Behl Y, Krothapalli P, Desta T, Roy S, Graves DT (2009). FOXO1 plays an important role in enhanced microvascular cell apoptosis and microvascular cell loss in type 1 and type 2 diabetic rats. *Diabetes*.

[47] Alikhani M, Roy S, Graves DT (2010). FOXO1 plays an essential role in apoptosis of retinal pericytes. *Molecular Vision*.

[48] Su D, Coudriet GM, Dae HK (2009). FoxO1 links insulin resistance to proinflammatory cytokine IL-1*β* production in macrophages. *Diabetes*.

[49] Fan W, Morinaga H, Kim JJ (2010). FoxO1 regulates Tlr4 inflammatory pathway signalling in macrophages. *EMBO Journal*.

[50] Alblowi J, Kayal RA, Siqueria M (2009). High levels of tumor necrosis factor-*α* contribute to accelerated loss of cartilage in diabetic fracture healing. *American Journal of Pathology*.

[51] Kayal RA, Siqueira M, Alblowi J (2010). TNF-*α* mediates diabetes-enhanced chondrocyte apoptosis during fracture healing and stimulates chondrocyte apoptosis through FOXO1. *Journal of Bone and Mineral Research*.

[52] Romeo G, Liu WH, Asnaghi V, Kern TS, Lorenzi M (2002). Activation of nuclear factor-*κ*B induced by diabetes and high glucose regulates a proapoptotic program in retinal pericytes. *Diabetes*.

[53] Griscavage JM, Wilk S, Ignarro LJ (1996). Inhibitors of the proteasome pathway interfere with induction of nitric oxide synthase in macrophages by blocking activation of transcription factor NF-*κ*B. *Proceedings of the National Academy of Sciences of the United States of America*.

[54] Yoon JC, Ng A, Kim BH, Bianco A, Xavier RJ, Elledge SJ (2010). Wnt signaling regulates mitochondrial physiology and insulin sensitivity. *Genes and Development*.

[55] Mantel C, Messina-Graham SV, Broxmeyer HE (2011). Superoxide flashes, reactive oxygen species, and the mitochondrial permeability transition pore: potential implications for hematopoietic stem cell function. *Current Opinion in Hematology*.

[56] Nishikawa T, Araki E (2007). Impact of mitochondrial ROS production in the pathogenesis of diabetes mellitus and its complications. *Antioxidants and Redox Signaling*.

[57] Diamond-Stanic MK, Henriksen EJ (2010). Direct inhibition by angiotensin II of insulin-dependent glucose transport activity in mammalian skeletal muscle involves a ROS-dependent mechanism. *Archives of Physiology and Biochemistry*.

[58] Fiory F, Lombardi A, Miele C, Giudicelli J, Beguinot F, van Obberghen E (2011). Methylglyoxal impairs insulin signalling and insulin action on glucose-induced insulin secretion in the pancreatic beta cell line INS-1E. *Diabetologia*.

[59] Matsumoto M, Pocai A, Rossetti L, DePinho RA, Accili D (2007). Impaired regulation of hepatic glucose production in mice lacking the forkhead transcription factor Foxo1 in liver. *Cell Metabolism*.

[60] Cheng Z, Guo S, Copps K (2009). Foxo1 integrates insulin signaling with mitochondrial function in the liver. *Nature Medicine*.

